# The contribution of granger causality analysis to our understanding of cardiovascular homeostasis: from cardiovascular and respiratory interactions to central autonomic network control

**DOI:** 10.3389/fnetp.2024.1315316

**Published:** 2024-08-08

**Authors:** Vincent Pichot, Christophe Corbier, Florian Chouchou

**Affiliations:** ^1^ Department of Clinical and Exercise Physiology, SAINBIOSE, Inserm U1059, Saint-Etienne Jean Monnet University, CHU Saint-Etienne, Saint-Etienne, France; ^2^ LASPI EA3059, Saint-Etienne Jean Monnet University, Roanne Technology University Institute, Roanne, France; ^3^ IRISSE Laboratory EA4075, University of La Réunion, UFR Science de ’Homme et de l’Environnement, Le Tampon, France

**Keywords:** homeostasis, cardiac, blood pressure, respiratory, Granger causality, autonomic nervous system

## Abstract

Homeostatic regulation plays a fundamental role in maintenance of multicellular life. At different scales and in different biological systems, this principle allows a better understanding of biological organization. Consequently, a growing interest in studying cause-effect relations between physiological systems has emerged, such as in the fields of cardiovascular and cardiorespiratory regulations. For this, mathematical approaches such as Granger causality (GC) were applied to the field of cardiovascular physiology in the last 20 years, overcoming the limitations of previous approaches and offering new perspectives in understanding cardiac, vascular and respiratory homeostatic interactions. In clinical practice, continuous recording of clinical data of hospitalized patients or by telemetry has opened new applicability for these approaches with potential early diagnostic and prognostic information. In this review, we describe a theoretical background of approaches based on linear GC in time and frequency domains applied to detect couplings between time series of RR intervals, blood pressure and respiration. Interestingly, these tools help in understanding the contribution of homeostatic negative feedback and the anticipatory feedforward mechanisms in homeostatic cardiovascular and cardiorespiratory controls. We also describe experimental and clinical results based on these mathematical tools, consolidating previous experimental and clinical evidence on the coupling in cardiovascular and cardiorespiratory studies. Finally, we propose perspectives allowing to complete the understanding of these interactions between cardiovascular and cardiorespiratory systems, as well as the interplay between brain and cardiac, and vascular and respiratory systems, offering a high integrative view of cardiovascular and cardiorespiratory homeostatic regulation.

## 1 Introduction

Claude Bernard was the first to propose in 1878 that life results in a continuous balance, for which “the fixity of the internal environment is the condition of free, independent life: the mechanism that allows it is the one that assures in the interior environment the maintenance of all the conditions necessary for the life of the elements” (Bernard, 1878). This notion of the internal environment – le milieu intérieur – and its interaction with the external environment will later become the pillar of the notion of homeostasis later proposed by Walter Cannon (Cannon, 1929; Cannon, 1932). Based on this, Walter Cannon proposed in 1929 the fruitful notion of homeostasis defined as the following: “Homeo, the abbreviated form of homoio, is prefixed instead of homo, because the former indicates “like” or “similar” and admits some variation, whereas the latter, meaning the “same”, indicates a fixed and rigid constancy. As in the branch of mechanics called “statics”, the central concept is that of a steady state produced by the action of forces” (Cannon, 1929). This definition has put interactions between systems at the center of organization and maintenance of multicellular life. This concept explains how an organism can maintain near constant internal conditions that allow, adapt and survive to changes and hostile external environments (Goldstein and Kopin, 2017; Billman, 2020).

Among the “constants” of the internal environments (Bernard, 1878), blood pressure (BP) could be considered as essential in cardiovascular homeostatic control and for adequate perfusion of tissues. Homeostatic regulation of BP is based on several mechanisms of different nature including hormonal (Ziaja et al., 2021), neuronal (Shivkumar et al., 2016) and mechanical controls (Saks et al., 2006). These mechanisms have to deal with internal constraints such as intrathoracic pressure related to respiratory modulations (Yasuma and Hayano, 2004) as well as with external environments and behavioral responses, including orthostatic challenges (Furlan et al., 2019), physical exercise (Fu and Levine, 2013), and activities related to high levels of cortical processes, such as during cognitive or emotional challenges (Ferraro et al., 2022).

This homeostatic regulation is mainly based on two controls: negative feedback and anticipatory feedforward mechanisms (Goldstein and Kopin, 2017). Negative feedback regulation is the main mechanism to maintain physiological homeostasis, as proposed by Walter Cannon: “When a factor is known which can shift a homeostatic state in one direction it is reasonable to look for automatic control of that factor or for a factor or factors having an opposing effect” (Cannon, 1929; Cannon, 1932). Anticipatory feedforward mechanisms are based on mediation by anticipatory adjustments in physiological systems related to knowledge of a previously experienced or instinctively recognized signal. The latter mechanism is more efficient than the former, as it decreases or eliminates the need for homeostatic adjustments that occur later (Goldstein and Kopin, 2017). Concerning the cardiovascular system and the regulation of BP, e.g., when going from a supine to a standing position, this anticipatory feedforward mechanism is illustrated by sympathoexicatory activation by the vestibular system, while the blood volume does not yet undergo fully its displacement towards the lower limbs by gravity (Carter and Ray, 2008). The sympathetic stimulation from arterial baroreflex receptors rather illustrates a feedback mechanism (Goldstein and Kopin, 2017).

**FIGURE 1:**
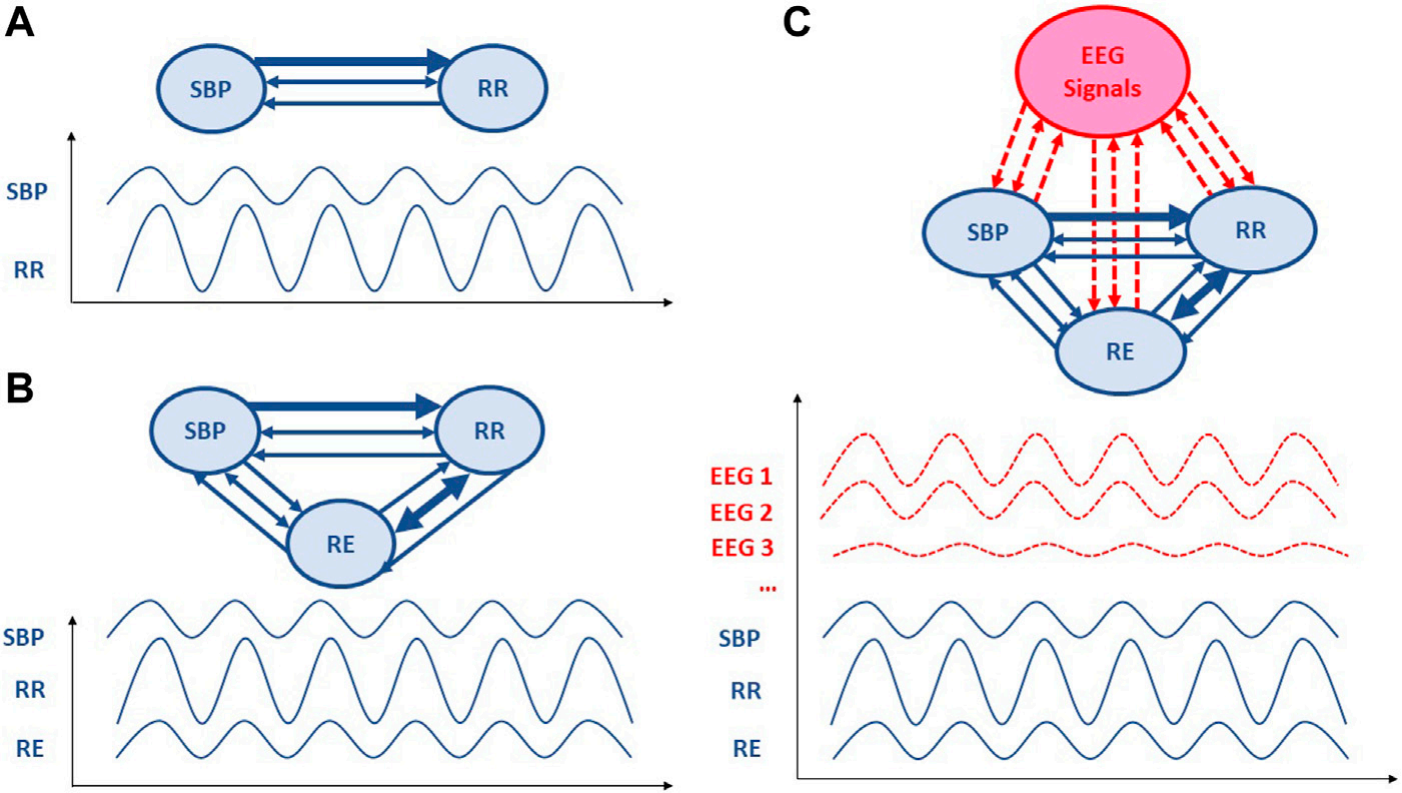
Schematic representation of Granger causality analysis applied to cardiovascular, respiratory and electroencephalographic signals. The direction of the arrows represents the direction of causality; two-way arrows mean that the two variables covary instantaneously. The thicker the arrow line, the more important is the causality link. **(A)** A bivariate model including only RR intervals (RRI) and systolic blood pressure (SBP) parameters. **(B)** Trivariate model also including respiratory (RE) parameter. **(C)** Further multivariate models would include brain regulations from electroencephalographic (EEG) signal or blood-oxygen-level dependent (BOLD) signal from functional *magnetic resonance imaging*.

These mechanisms play a role in generating fluctuations in cardiovascular and respiratory parameters, including RR intervals (RRI), systolic blood pressure (SBP) and respiration (RE), as they evolve over time (Figure 1). This observation dates back to the beginnings of modern medicine, almost 300 years ago, when variations in heart rhythm were measured and associated with variations with BP and RE (Hales and Woodward, 1733; Ludwig, 1847). Our understanding and use of these oscillations in research on the cardiovascular and respiratory systems advanced from the 1960s when these oscillations were considered as clinically and physiologically relevant (Hon and Lee, 1963; Murray et al., 1975; Wolf et al., 1978). Among the first, Hon and Lee (1963) reported that fetal stress was preceded by a transitory decrease in RRI; Murray et al. (1975) reported that short-term changes in RRI were altered in diabetic patients with a diagnosis of neuropathy; and Wolf et al. (1978) demonstrated for the first time a relationship between RRI variability and mortality following myocardial infarction. Since these pioneering studies, the field has rapidly expanded and the question of physiological interpretation of these oscillations arose. In the early 1970s, several groups applied power spectral analysis to investigate the physiological basis that compose these periodic variations in RRI and SBP (Hyndman et al., 1971); and later, particularly pharmacological studies, enabled us to better understand these rhythms (Akselrod et al., 1981; Pomeranz et al., 1985). It is now clearly established from pharmacological blockades of sympathetic and parasympathetic receptors that RRI variability above 0.05 Hz is mainly due to change in autonomic control to the sinoatrial node. A certain number of mathematical methods make it possible to study in a noninvasive way these variations from a simple electrocardiographic (ECG) recording, belonging to the fields of temporal, geometric, frequency or nonlinear analysis (European Society of Cardiology and the North American Society of Pacing and Electrophysiology, 1996). For frequency domain, high frequency (0.15–0.5 Hz) oscillations depend mainly on the parasympathetic system and changing levels of vagal nerve activity; while low frequency (0.04–0.15 Hz) oscillations may be mediated by either cardiac parasympathetic or sympathetic activities (Eckberg, 2000). Very low frequency fluctuations below ∼0.04 Hz may also be mediated by change in autonomic control as well as in plasma hormones or other non-autonomic influences (Saul and Valenza, 2021). Concerning the vascular system, pharmacological blockage revealed that only low frequency power of SBP is mainly under sympathetic control (Pagani et al., 1986), although indirect parasympathetic modulations may influence low frequency power of SBP (Fontolliet et al., 2018). Additionally, cross-spectral power density and cross-correlation analyses allowed to study two signals associated with a given time shift and/or frequency. Applied to RRI, SBP and RE signals, they make it possible to study the interactions between cardiac, vascular and respiratory systems (Porta and Faes, 2013). Two mechanisms have emerged as fundamental: 1) respiratory sinus arrhythmia resulting from an interaction between the respiratory and cardiac systems (Yasuma and Hayano, 2004); and 2) baroreflex loop based on interaction between the cardiac and vascular systems (Wehrwein and Joyner, 2013). These interactions are fundamental for the adaptation of the organism to external and internal constraints, as illustrated by their ability to predict morbidity and mortality (Tsuji et al., 1994; Dekker et al., 1997; Gerritsen et al., 2001; Hämmerle et al., 2020).

However, using RRI and SBP variabilities remain complex to interpret and does not allow a completely satisfactory exploration of the interactions between the different physiological systems involved. Indeed, these methods present some difficulties. Firstly, strong criticism has emerged regarding the ability of low frequency RRI to approach cardiac sympathetic tone (Billman, 2013; Heathers, 2014; La Rovere et al., 2020). Autonomic influence on the spectrum of RRI is essentially parasympathetic and the contribution of sympathetic modulation in RRI variability is very modest (Akselrod et al., 1981; Pomeranz et al., 1985; Eckberg, 2000), resulting in a lack of precision to quantify the cardiac sympathetic tone (Billman, 2013). Secondly, interactions between the cardiac, vascular and respiratory systems are more complex; and other mechanisms such as the Windkessel effect or mechanical interactions between pulmonary tissue and heart volume are not taken into account in these analyses (Centracchio et al., 2022). Moreover, cardiovascular parameters are characterized by complexes and beat-to-beat interactions within the cardiovascular system, from heart to vascular (Westerhof et al., 2009) and from cranial and vascular systems to heart control (Beiner et al., 1997; Wehrwein and Joyner, 2013), with other biological systems such as the respiratory system (Yasuma and Hayano, 2004), as well as with external constraints through anticipatory feedforward controls such as pain (Chouchou et al., 2011; Fauchon et al., 2018). Thirdly, cross-spectral power density and cross-correlation analyses do not account for either the direction of the interactions or the temporal sequence of the activation of the mechanisms contributing to the observed association (Porta and Faes, 2013; Schulz et al., 2013; Müller et al., 2016).

Thus the need has arisen to use new closed loop models applied to cardiovascular and respiratory signals in order to deepen our knowledge of the underlying physiological regulatory processes and to possibly obtain better indices for predicting morbidity and mortality (Porta and Faes, 2013; Schulz et al., 2013; Müller et al., 2016). For this, the analysis of causal relationships within dynamic systems has become more and more used in the physiological field and seems to be suited to capture complex interactions between time series such as RRI, SBP and RE, allowing the detection and quantification of the strength and direction of couplings. The most studied and promising approach is based on the notion of Granger causality (GC), implying that if one time series has a causal influence on a second time series, then the knowledge of the past of the first time series is useful to predict future values of the second time series (Granger, 1969).

In this review, the aim is to introduce GC and provide an overview of the key GC tools available. These tools can help enhance our comprehension of physiological processes underlying cardiovascular homeostasis. For this, 1) we describe approaches based on GC applied to detect couplings between time series, especially focusing on cardiovascular and respiratory parameters; we present their theoretical background and their usefulness for detecting causality. Then 2) we discuss results of coupling analyses in cardiovascular and cardiorespiratory studies, including those comparing the results of these presented methods and those of traditional approaches. Lastly, 3) we suggest potential approaches to optimize their utilization in relation to these physiological parameters and why these analyses naturally lead to an interest in the heart-brain relationship. To address these questions, this review is based on a search of Medline and the Cochrane Library up to March 2024. Keywords used to research cardiovascular homeostasis were “Granger causality” associated with “cardiac”, “heart”, “respiratory”, or “blood pressure”. To fully understand this review, two precautions are necessary: 1) we have deliberately limited this review to articles using Granger’s temporal and frequency methods applied to cardiovascular and respiratory signals; other methods dealing with causality, in the sense of transfer entropy for example, are not covered in the review; 2) we have considered SBP and its changes as representative of the vascular system, despite the fact that we are well aware that other parameters can influence SBP, notably RRI. Throughout this review, it should be kept in mind that the three physiological signals of RRI, BP and RE are highly interconnected, hence the value of using these Granger methods.

## 2 Causality: generalities and methods

### 2.1 Generalities

Causality is a generic term meaning cause-effect relationships between systems, subsystems, processes or phenomena. In our research on cardiovascular homeostasis, we investigated physiological processes by focusing on key signals, including RRI, SBP and RE (Figure 1). Causality analysis between RRI, SBP and RE can reveal mechanisms governing RRI ↔ SBP ↔ RE dynamic interactions (Cohen and Taylor, 2002; Porta et al., 2013b). Granger’s definition of causality in multivariate stochastic processes provided a framework for estimating causality in time series (Granger, 1980). Given a set of M signals, Ω , describing the behavior of a system, the time series $X_{j}\left(t\right)$ causes $X_{i}\left(t\right)$ in Ω if the inclusion of past observations of $X_{j}\left(t\right)$ reduces the prediction error of $X_{i}\left(t\right)$ . In the literature, GC is described using time-domain and frequency-domain approaches. The latter has been widely developed in a strictly causal multivariate (MVAR) model by Kaminski and Blinowska (1991), introducing a new measure from directed transfer function (DTF) (Baccala et al., 1998; Baccalá and Sameshima, 2001) with directed coherence (DC) and partial directed coherence (PDC). Faes and Nollo (2010) proposed to extend the MVAR representation by introducing extended directed coherence (eDC) and extended partial directed coherence (ePDC).

The GC time series approach is separated into two MVAR representations: strictly causal and extended. The strictly causal approach is the origin of GC (cGC) where a MVAR only contains the past terms $\left(k>0\right)$ . The causality from i to j is given by ${cGC}_{i\to j}=\ln\left(\frac{var{\bar{U}}_{j}\left(t\right)}{varU_{j}\left(t\right)}\right)$ , where $var{\bar{U}}_{j}\left(t\right)$ is the variance of the restricted regression and $varU_{j}\left(t\right)$ the variance of the unrestricted regression. Porta et al. (2013a) defined the *directionality index* as $DI_{ij}={cGC}_{i\to j}-{cGC}_{j\to i}$ . $DI_{ij}>0$ indicates that the causal direction from $X_{j}\left(t\right)$ to $X_{i}\left(t\right)$ is prevalent over the reverse one, while $DI_{ij}<0$ is the opposite situation. The directionality index is capable to give dominant causality. For example, $DI_{ij}>0$ or $DI_{ij}<0$ does not exclude bidirectional interactions. $DI_{ij}$ close to 0 might indicate:

(i) A full uncoupling between Xi(t) and Xj(t)
(ii) Closed loop interactions between Xi(t) and Xj(t)with no dominance of Xi(t) or Xj(t)
(iii) Synchronization between Xi(t) and Xj(t)

In its original formulation, GC results from strictly causal MVAR representation of the observed processes and is described in terms of linear regressions. This presupposes that the considered model has the full interaction structure of the observed processes. However, if the causal interpretation is not sufficient from regression coefficients, GC may measure misleading patterns of causality. Actually, strictly causal MVAR interpretation is a restricted form of the GC approach since this formulation only includes time-lagged and not instantaneous effects. The consequence is to propose a complete representation including instantaneous effects $\left(k=0\right)$ through a new GC approach named extended GC (eGC). Studies (Hyvärinen et al., 2010; Schiatti et al., 2015) have demonstrated the adverse impact of neglecting instantaneous effects on causality analysis based on the MVAR model, providing theoretical arguments showing that the omission of zero-lag correlations from the model can change drastically the values of time-lagged coefficients, and thus of the causality measures based on the model. As a possible solution, these studies proposed the utilization of an extended MVAR model including both instantaneous and lagged effects. The extended model was devised combining a classical MVAR model and a model of the instantaneous causal interactions among the observed variables commonly known as a structural equation model (SEM). Model estimation was performed using conventional least-squares regression for the VAR part, and a more sophisticated method called linear non-Gaussian acyclic model (LiNGAM) (Shimizu et al., 2006) that exploits non-Gaussianity to address the known identifiability problem of SEM models. The extended causality from i to j is given by ${eGC}_{i\to j}=\ln\left(\frac{var{\bar{W}}_{j}\left(t\right)}{varW_{j}\left(t\right)}\right)$ , where $var{\bar{W}}_{j}\left(t\right)$ is the variance of the restricted regression and $varW_{j}\left(t\right)$ the variance of the unrestricted regression.

The causality from frequency approach has been widely investigated for two representations: strictly causal and extended MVAR. Strictly causal MVAR representation provided measurements such as DC and PDC (Baccala et al., 1998; Baccalá and Sameshima, 2001). In the extended representation, eDC and ePDC have been presented (Faes and Nollo, 2010); therefore, these measures indicate the following.

(i) the direct causality from PDC
(ii) the extended direct causality from ePDC
(iii) the causality from DC
(iv) the extended causality from eDC

For strictly causal MVAR representation, consider $Z_{j}=\left\{X_{l},l=1,\ldots ,M,l\neq j\right\}$ , where $X_{l}=\left\{\begin{array}{ccc} X_{l}\left(t-1\right) & \ldots & X_{l}\left(t-d\right) \end{array}\right\}$ the set of the past values of all processes except $X_{j}$ . *Direct causality* $X_{j}\left(t\right)\to X_{i}\left(t\right)$ exists if the prediction of $X_{i}\left(t\right)$ based on $Z_{j}$ and $X_{j}$ is better than the prediction of $X_{i}\left(t\right)$ solely based on $Z_{j}$ . *Causality* $X_{j}\left(t\right)\Rightarrow X_{i}\left(t\right)$ exists if a cascade of direct causality relations $X_{j}\left(t\right)\to X_{m}\left(t\right)\ldots \to X_{i}\left(t\right)$ occurs for at least one value m in the set $\left(1,\ldots ,M\right)$ . Analogously, extended MVAR representation denotes ${\hat{Z}}_{j}=\left\{{\hat{X}}_{l},l=1,\ldots ,M,l\neq j\right\}$ , where ${\hat{X}}_{l}=\left\{\begin{array}{ccc} X_{l}\left(t\right)X_{l}\left(t-1\right) & \ldots & X_{l}\left(t-d\right) \end{array}\right\}$ the set of the past values of all processes except $X_{j}$ . *Extended direct causality* $X_{j}\left(t\right)\overset{´}{\to }X_{i}\left(t\right)$ exists if the prediction of $X_{i}\left(t\right)$ based on ${\hat{Z}}_{j}$ and ${\hat{X}}_{j}$ is better than the prediction of $X_{i}\left(t\right)$ solely based on ${\hat{Z}}_{j}$ . *Extended causality* $X_{j}\left(t\right)\overset{´}{\Rightarrow }X_{i}\left(t\right)$ exists if a cascade of extended direct causality relationship $X_{j}\left(t\right)\overset{´}{\to }X_{m}\left(t\right)\ldots \overset{´}{\to }X_{i}\left(t\right)$ occurs for at least one value m in the set $\left(1,\ldots ,M\right)$ . Expressions of DC (resp. eDC) are established from coherence (Coh) and PDC (resp. ePDC) from partial coherence (PCoh). Below, mathematical aspects for GC in time-domain and causality-based coherence in frequency domain are described.

### 2.2 Different methods to explore causality

In this part, we present a nonexhaustive description of main tools and mathematical aspects applied in physiology in order to yield for the user a basic knowledge. The central subject concerns causality. We will try to present a limited mathematical development that only focuses on GC and causality-based coherence used in the field of cardiovascular and respiratory interaction explorations (see Supplementary Tables S1, S2).

#### 2.2.1 Causality based granger approach in time domain

Assessing causality from the Granger approach is the most popular method, including the multivariate GC (MVGC) toolbox (Barnett and Seth, 2014). GC or classical GC (cGC) is a popular tool for the user for assessing the presence of directional interactions between two time series of a multivariate data set (Wiener, 1956; Granger, 1969). However, cGC only includes the time-lagged effects between processes. In respiratory and cardiovascular physiology, significant instantaneous effects are present (Faes, 2014; Schiatti et al., 2015). Subsequently, cGC may lead to an incomplete description of the “real” phenomenon between processes. As a possible solution, the utilization of an extended model accounting for both instantaneous and lagged effects has been proposed. This modelling is named eGC. Both methods are described below.

##### 2.2.1.1 Classical GC

In 1969, Granger introduced a GC approach in terms of multivariate linear regression modeling (Granger, 1969). For example, according to GC, $X_{j}\left(t\right)$ causes $X_{i}\left(t\right)$ if the inclusion of (Faes, 2014) past observations of $X_{j}\left(t\right)$ reduces the prediction error of $X_{i}\left(t\right)$ in a linear regression model of $X_{i}\left(t\right)$ and $X_{j}\left(t\right)$ , as compared to a model including only previous observations of $X_{i}\left(t\right)$ . Let $X\left(t\right)$ be the signal vector, such that $X\left(t\right)={\left(\begin{array}{ccc} X_{1}\left(t\right) & \ldots & X_{M}\left(t\right) \end{array}\right)}^{T}$ . The VAR model of order d is:

$$X\left(t\right)=\sum_{k=1}^{d}A\left(k\right)X\left(t-k\right)+U\left(t\right)$$ (1)

where $U\left(t\right)$ are model residuals, and $A\left(k\right)$ are M×M coefficients matrices with elements $A_{ji}\left(k\right)$ .

To compute cGC from the *i*th process $X_{i}\left(t\right)$ to the *j*th process $X_{j}\left(t\right)$ , two linear regressions are considered starting from (Eq. 1). First, $X_{j}\left(t\right)$ is described in terms of the past of all processes in $X\left(t\right)$ by means of a so called *unrestricted* regression, such that:

$$X_{j}\left(t\right)=\sum_{k=1}^{d}A_{j}\left(k\right)X\left(t-k\right)+U_{j}\left(t\right)$$

with $A_{j}\left(k\right)$ the *j*th row of $A\left(k\right)$ . Then $X_{j}\left(t\right)$ is described in terms of the past of all processes in $X\left(t\right)$ except $X_{i}\left(t\right)$ by means of a so called “restricted” regression:

$$X_{j}\left(t\right)=\sum_{k=1}^{d}{\hat{A}}_{j}\left(k\right)X^{i}\left(t-k\right)+{\hat{U}}_{j}\left(t\right)$$

where $X^{i}\left(t\right)={\left(\begin{array}{ccc} X_{1}\left(t\right)\ldots & X_{i-1}\left(t\right)X_{i+1}\left(t\right)\ldots & X_{M}\left(t\right) \end{array}\right)}^{T}$ denotes the original vector process devoid of the *i*th scalar process. Moreover, ${\hat{A}}_{j}$ and the residuals ${\hat{U}}_{j}$ of the restricted regression are different than $A_{j}$ and the residuals $U_{j}$ of the unrestricted regression. The cGC measure is given by:

$${cGC}_{i\to j}=\ln\left(\frac{var{\hat{U}}_{j}\left(t\right)}{varU_{j}\left(t\right)}\right)$$

The model of 1) is a strictly causal model that describes only the time-lagged interactions between the processes. Thus cGC is computed without considering the instantaneous effects among observed time series. This approach is applied in several studies concerning the interactions between cardiovascular and respiratory systems in heathy volunteers (Porta et al., 2013b) and clinical populations (Riedl et al., 2010; Bassani et al., 2012; Bassani et al., 2013; Bassani et al., 2014; Zamunér et al., 2017).

##### 2.2.1.2 Extended GC

The possible presence of zero-lag effects is known to also have an impact on the time-lagged effects (Hyvarinen et al., 2008; Hyvärinen et al., 2010; Faes, 2014; Schiatti et al., 2015), and hence may affect the reliability of the observed GC patterns. To overcome this problem, an extended GC combining both instantaneous and lagged effects is given by:

$$X\left(t\right)=\sum_{k=0}^{d}B\left(k\right)X\left(t-k\right)+W\left(t\right)$$ (2)

where $W\left(t\right)$ are model residuals, and $B\left(k\right)$ are M×M coefficients matrices with elements $B_{ji}\left(k\right)$ . The zero-lag effects are contained in matrix $B\left(0\right)$ of 5): allowing an effect at lag zero between two processes, where $X_{i}\left(t\right)$ and $X_{j}\left(t\right)$ correspond to set either $B_{ij}\left(0\right)\neq 0$ or $B_{ji}\left(0\right)\neq 0$ . The coefficients cannot both be nonzero because the presence of a pairwise loop would make the extended model unidentifiable (Shimizu et al., 2006; Hyvärinen et al., 2010; Faes et al., 2013).

To compute eGC from the *i*th process $X_{i}\left(t\right)$ to the *j*th process $X_{j}\left(t\right)$ , two linear regressions are considered starting from (Eq. 5). First, $X_{j}\left(t\right)$ is described in terms of the present and past of all processes in $X\left(t\right)$ by means of a so called “unrestricted” regression, such that:

$$X_{j}\left(t\right)=\sum_{k=0}^{d}B_{j}\left(k\right)X\left(t-k\right)+W_{j}\left(t\right)$$ (3)

with $B_{j}\left(k\right)$ the *j*th row of $B\left(k\right)$ . Then $X_{j}\left(t\right)$ is described in terms of the present and past of all processes in $X\left(t\right)$ except $X_{i}\left(t\right)$ by means of the so called “restricted” regression:

$$X_{j}\left(t\right)=\sum_{k=0}^{d}{\hat{B}}_{j}\left(k\right)X^{i}\left(t-k\right)+{\hat{W}}_{j}\left(t\right)$$ (4)

where $X^{i}\left(t\right)={\left(\begin{array}{ccc} X_{1}\left(t\right)\ldots & X_{i-1}\left(t\right)X_{i+1}\left(t\right)\ldots & X_{M}\left(t\right) \end{array}\right)}^{T}$ denotes the original vector process devoid of the *i*th scalar process. The cGC measure is given by:

$${eGC}_{i\to j}=\ln\left(\frac{var{\hat{W}}_{j}\left(t\right)}{varW_{j}\left(t\right)}\right)$$ (5)

The estimation of eGC requires identifying the presence and causal direction of the zero-lag effects between the observed processes to be incorporated into the regression models (6) and (7). Therefore, to accomplish this task a two-step procedure base is used: first on estimating the existence of zero-lag correlations in an undirected sense, and then on finding their directions using pairwise measures of non-Gaussianity (Hyvärinen and Smith, 2013). The matrix $B\left(0\right)$ is related to instantaneous effects. This approach has been applied in several studies concerning the interactions between cardiovascular and respiratory systems in healthy volunteers (Schiatti et al., 2015).

##### 2.2.1.3 Causal direction or direction of effects

From a causality point of view and before estimating the MVAR model, an important step is required by determining measures of the causal direction. We present main tools used by the community based on pairwise likelihood ratios (Hyvärinen and Smith, 2013).

Denote the two observed random variables by x and y . Assume these variables are non-Gaussian standardized to zero mean and unit variance. The goal is to distinguish two causal models: y=ρx+d and x=ρy+e , where disturbances d is independent of x and e independent of y . The parameter ρ is the correlation coefficient. Hyvärinen and Smith (2013) showed that the likelihood ratio R normalized by $\frac{1}{N}$ is:

$$R=\frac{1}{N}\sum_{t=1}^{N}\left[\log p_{x}\left(x_{t}\right)+G_{d}\left(\frac{y_{t}-\rho x_{t}}{\sqrt{1-\rho^{2}}}\right)-\log p_{y}\left(y_{t}\right)\right.\left.-G_{e}\left(\frac{x_{t}-\rho y_{t}}{\sqrt{1-\rho^{2}}}\right)\right]$$

where $G_{d}$ and $G_{e}$ are the standardized log-pdf’s of the residuals when regressing y on x and x on y , respectively.

The rule of causal direction is chosen as:

• x→y if R is positive

• y→x if R is negative

However, the choice of the four log-pdf’s $\left(\log p_{x}\left(x_{t}\right)\right.$ , $\log p_{y}\left(y_{t}\right)$ , $G_{d}$ , and $G_{e}$ ) can be difficult for the modelling. Therefore, various parametric approximations have been given by Hyvärinen and Smith (2013).

#### 2.2.2 Causality based coherence approach in frequency domain

Notions of causality are commonly formalized in the context of a MVAR representation of time series in order to allow time- and frequency-domain pictures. Several frequency domain measures of causality have been introduced. Actually, measures to quantify causality in the frequency domain have been proposed from a strictly causal MVAR representation (see (1)): DTF (Kaminski and Blinowska, 1991), DC (Baccala et al., 1998), and PDC (Baccalá and Sameshima, 2001). Faes and Nollo (2010) have extended DC and PDC measures to extended causal MVAR representation (see (5)) to provide eDC and ePDC. A synthesis of all measures of causality and coupling has been proposed in Faes et al. (2011).

Denoting in the strictly causal MVAR interpretation $Z_{j}=\left\{X_{l},l=1,\ldots ,M,l\neq j\right\}$ , where $X_{l}=\left\{\begin{array}{ccc} X_{l}\left(t-1\right) & \ldots & X_{l}\left(t-d\right) \end{array}\right\}$ the set of the past values of all processes except $X_{j}$ . *Direct causality* $X_{j}\left(t\right)\to X_{i}\left(t\right)$ exists if the prediction of $X_{i}\left(t\right)$ based on $Z_{j}$ and $X_{j}$ is better than the prediction of $X_{i}\left(t\right)$ solely based on $Z_{j}$ . *Causality* $X_{j}\left(t\right)\Rightarrow X_{i}\left(t\right)$ exists if a cascade of direct causality relations $X_{j}\left(t\right)\to X_{m}\left(t\right)\ldots \to X_{i}\left(t\right)$ occurs for at least one value m in the set $\left(1,\ldots ,M\right)$ . DC and PDC are measures of the direct directed interaction occurring from $X_{j}\left(t\right)$ to $X_{i}\left(t\right)$ at the frequency f . PDC measures the direct causality and DC the causality.

##### 2.2.2.1 Coherence in strictly causal MVAR representation

The Fourier transform (FT) of 1) is $X\left(f\right)=A\left(f\right)X\left(f\right)+U\left(f\right)$ and from $H\left(f\right)={\left[I-A\left(f\right)\right]}^{-1}={\hat{A}\left(f\right)}^{-1}$ the power spectral density (psd) is $S\left(f\right)=H\left(f\right)\Sigma H^{†}\left(f\right)$ , where $\left(†\right)$ is the Hermitian transpose and $\Sigma =cov\left(U\left(f\right)\right)=\left(\sigma_{ij}\right)$ the variance/covariance matrix of $U\left(f\right)$ . Let $S_{ij}\left(f\right)$ and $H_{ij}\left(f\right)$ be components of $S\left(f\right)$ and $H\left(f\right)$ , respectively. The DC $\gamma_{ij}\left(f\right)$ is given by:

$$\gamma_{ij}\left(f\right)=\frac{\sigma_{j}H_{ij}\left(f\right)}{\sqrt{\sum_{m=1}^{M}\sigma_{m}^{2}{\left|H_{I}\left(f\right)\right|}^{2}}}$$

and the PDC by:

$$\gamma_{ij}\left(f\right)=\frac{\frac{1}{\sigma_{i}}{\hat{A}}_{ij}\left(f\right)}{\sqrt{\sum_{m=1}^{M}\frac{1}{\sigma_{m}^{2}}{\left|{\hat{A}}_{mj}\left(f\right)\right|}^{2}}}$$

This approach has been applied in several studies concerning the interactions between cardiovascular and respiratory systems in heathy volunteers (Javorka et al., 2017) and clinical populations (Lachert et al., 2019).

##### 2.2.2.2 Coherence in extended MVAR representation

The FT of 5) is $X\left(f\right)=B\left(f\right)X\left(f\right)+W\left(f\right)$ and from $G\left(f\right)={\left[I-B\left(f\right)\right]}^{-1}={\hat{B}\left(f\right)}^{-1}$ the psd is $S\left(f\right)=G\left(f\right)\Lambda G^{†}\left(f\right)$ , where $\Lambda =cov\left(W\left(f\right)\right)=\left(\lambda_{ij}\right)$ the variance/covariance matrix of $W\left(f\right)$ . Let $S_{ij}\left(f\right)$ and $G_{ij}\left(f\right)$ be components of $S\left(f\right)$ and $G\left(f\right)$ , respectively. The eDC $\xi_{ij}\left(f\right)$ is given by:

$$\xi_{ij}\left(f\right)=\frac{\lambda_{j}G_{ij}\left(f\right)}{\sqrt{\sum_{m=1}^{M}\lambda_{m}^{2}{\left|G_{I}\left(f\right)\right|}^{2}}}$$

and the ePDC by:

$$\chi_{ij}\left(f\right)=\frac{\frac{1}{\lambda_{i}}{\hat{B}}_{ij}\left(f\right)}{\sqrt{\sum_{m=1}^{M}\frac{1}{\lambda_{m}^{2}}{\left|{\hat{B}}_{mj}\left(f\right)\right|}^{2}}}$$

This approach has been applied in several studies concerning the interactions between cardiovascular and respiratory systems in heathy volunteers (Faes and Nollo, 2010) and clinical populations (Charleston-Villalobos et al., 2019; Reulecke et al., 2019).

## 3 Applications of cardiovascular and cardiorespiratory coupling analyses

### 3.1 First studies using GC applied in cardiovascular field

Porta et al. (2002) followed by Nollo et al. (2005) were the first to apply these methods to the cardiovascular and respiratory systems (Supplementary Tables S1, S2). These first studies are already very complete, carried out in dogs, patients and healthy volunteers, demonstrating the interests of the approach (Porta et al., 2002; Nollo et al., 2005). Porta et al. (2002) applied GC from coherence functions of RRI and noninvasive finger SBP monitoring. Applied to 12 instrumented conscious dogs (4 dogs with total baroreceptor denervation), 7 heart transplant recipients and 7 matched healthy subjects, they observed that the causal direction from RRI to SBP predominated in the HF band in dogs, in absence of any effect of baroreceptive denervation. In heart transplant recipients, they observed only coupling from the RRI to SBP direction in the LF bandwidth, but in both directions in HF bandwidth in the matched healthy subjects. In the same way, based also on causal coherence between RRI and SBP in 15 healthy young subjects, Nollo et al. (2005) reported predominance of the causal direction from RRI to SBP in the LF band but balanced in the HF band during supine at rest. During tilt, the causal direction from SBP to RRI increased and predominated in both LF and HF bands. These first studies showed that this type of analysis makes it possible to study the main mechanical or neuronal interactions between the cardiac and vascular systems: these results were interpreted as the Windkessel and Starling effects and are captured by the interaction from RRI to SBP; whereas the interaction from SBP to RRI was considered as from feedback baroreflex activations, and interactions from RRI to SBP as non-baroreflex mechanisms including neural and mechanical (Windkessel and Starling effects) controls (Fuchs and Smith, 2001; Westerhof et al., 2009). More recently, Krohova et al. have clearly demonstrated the causal link between SBP and peripheral vascular resistance (Krohova et al., 2020). It is interesting to note here that traditional arterial baroreflex or autonomic analyses show an activation of parasympathetic baroreflex sensitivity in the supine position and a sympathetic activation by the tilt test (Wehrwein and Joyner, 2013). Here, the causality undoubtedly informs us about the quantity of interaction between these two systems but not about the gain or the nature of these interactions between these signals (Porta and Faes, 2013; Schulz et al., 2013; Müller et al., 2016). As a result, the two indices derived from the traditional and GC methods do not provide the same information but are complementary.

GC analysis methods have been progressively adapted to the constraints of physiological signals (Faes et al., 2010; Porta et al., 2013b). In this way, a zero-lag effect has been demonstrated, i.e., an instantaneous causality effect visible more particularly on the BP→RRI index, due to the fast response of the parasympathetic arm within a cardiac cycle and RE→RRI and RE→BP relationships, due to the rapid mechanical effect of respiration motion on cardiac filling (Hyvarinen et al. 2008; Faes et al., 2010; Faes, 2014; Reulecke et al., 2019). This methodological aspect is discussed in Section 4.2. Readers should be aware that early studies of GC in the cardiovascular and cardiorespiratory fields did not take this zero-lag effect into account and may sometimes present slightly different results from more recent studies for certain indices. Finally, despite their obvious interests, these studies raise questions about the role of respiration in this closed loop regulation and, therefore, the question of a bi- or trivariate modelling. This was subsequently addressed in numerous studies (Faes et al., 2006; Faes et al., 2011; Faes and Nollo, 2010; Riedl et al., 2010; Porta et al., 2012) and is discussed below (Section 4.4.).

Since these pioneering works, we have identified 42 additional studies applying this principle of GC in the field of cardiovascular and respiratory interactions, where the population sample which can range from 1–100 healthy volunteers (Faes et al., 2006; Faes et al., 2008; Faes and Nollo, 2010; Porta et al., 2012; Porta et al., 2012; Porta et al., 2013b; Porta et al., 2014a; Porta et al., 2014b; Faes et al., 2013; Faes et al., 2015b; Porta et al., 2015; Song et al., 2015; Javorka et al., 2017; Porta et al., 2018; Saleem et al., 2018; Lachert et al., 2019; Corbier et al., 2020; Nuzzi et al., 2021) or increasingly in diverse populations of patients (Porta et al., 2002; Porta et al., 2013a; Porta et al., 2013b; Bassani et al., 2014; Faes et al., 2015b; Javorka et al., 2015; Schiatti et al., 2015; Zamunér et al., 2015; Bose et al., 2017; Zamunér et al., 2017; Charleston-Villalobos et al., 2019; Reulecke et al., 2019; Schulz et al., 2019; Stramaglia et al., 2021) (see Supplementary Tables S1, S2). The trivariate approach is increasingly used to integrate the respiratory signal in order to not only to draw up the more complete picture possible of the interactions of these systems but also to properly analyze the BP-RRI relationship. These multivariate approaches were also supplemented by the introduction of other parameters such as cerebral blood flow velocity (Faes et al., 2015b; Schiatti et al., 2015; Saleem et al., 2018), cerebral oxygenation (Song et al., 2015), peripheral blood oxygen saturation (Bose et al., 2017), peripheral vascular resistance (Krohova et al., 2020) and left ventricular ejection time (Javorka et al., 2015), QT interval (Porta et al., 2015) or end-tidal CO_2_ (Saleem et al., 2018), as well as by electroencephalographic (EEG) signals in particular (Schulz et al., 2019; Hartmann et al., 2021).

### 3.2 Comparison with pharmacological studies

Among the studies that have made it possible to better understand how GC can improve our understanding of physiological system interactions, GC was applied to pharmacological maneuvers allowing blocking the sympathetic and/or parasympathetic influence on the sinoatrial node. Indeed, the blockage of the cardiovascular autonomic nervous system can allow to highlight a neuronal contribution in these cardiovascular and respiratory interactions (Pomeranz et al., 1985; Pichot et al., 1999).

Thus Porta et al. (2013a) applied trivariate GC in time domain on RRI, SBP and RE in 9 healthy volunteers under autonomic pharmacological blockages. Atropine, propranolol and clonidine were administered to block muscarinic receptors, β-adrenergic receptors and centrally sympathetic outflow, respectively. They observed that, as expected, a lengthening of the RRI and an increase in SBP in atropine and atropine + propranolol conditions without major changes in respiratory frequency consistent with previous studies (Pomeranz et al., 1985; Pichot et al., 1999), and propranolol and clonidine decreased RRI, whereas clonidine decreased SBP. Concerning causality, they reported that firstly RRI and SBP interacted at rest in a closed loop with a dominant causal direction from RRI to SBP, and that pharmacological blockades did not alter the bidirectional closed loop interactions between RRI and SBP; whereas atropine reduced the dominance of the causal direction from RRI to SBP. These results indicated that Windkessel and Starling effects may dominate the interaction from RRI to SBP, but this result points out a contribution of parasympathetic cardiac control that may favor cardiac filling (Fuchs and Smith, 2001; Westerhof et al., 2009). Saleem et al. (2018) extended these results by studying the relationship between very slow components of SBP, RRI and cerebral blood flow velocity fluctuations using α1-adrenergic blockade in healthy volunteers. They observed bidirectional interaction between cerebral blood flow velocity and BP, consistent with the Cushing mechanism, regulating cerebral flow based on sympathetic control (Beiner et al., 1997; Ayling, 2002). In Porta et al. (2013a), at baseline, bidirectional interactions between RRI and RE were frequently found and this closed loop relationship was unmodified by the administration of drugs, illustrating nonneural interactions from RE to RRI, and potentially related to respiratory changes in intrathoracic pressure (Berntson et al., 1993; Hayano et al., 1996). Conversely, the result of causality from RRI to RE may be related to central respiratory drive (Porta et al., 2013b). Finally, unidirectional interactions from RE to SBP were often found at baseline, but atropine induced frequently an uncoupling between RE and SBP; whereas clonidine favored bidirectional interactions, pointing out an indirect parasympathetic contribution to the RE-SBP relationship through cardiac and large vessel fillings reversed by clonidine (Porta et al., 2013b).

These results prove that trivariate time domain measures of GC can contribute to the description of cardiovascular control by suggesting the temporal direction of the interactions and by separating different causality schemes. Causality analysis provides complementary information of classical mathematical tools including arterial baroreflex analysis, RRI and SBP variabilities that inform about baroreflex gain and autonomic/reactivity of the autonomic nervous system (Malliani et al., 1991; Laude et al., 2004; Saul and Valenza, 2021). Finally, expanding to encompass other cardiovascular and respiratory parameters can provide a more comprehensive perspective on cardiovascular regulation (Faes et al., 2015b; Javorka et al., 2015; Porta et al., 2015; Song et al., 2015; Bose et al., 2017; Saleem et al., 2018).

### 3.3 Comparison with autonomic measurements

To gain a deeper insight into the underlying mechanisms influencing the outcomes of GC and its relevance in clinical populations, several studies have compared GC with traditional physiological indices (see Supplementary Tables S1, S2). In essence, these studies conducted causality analyses, which were broadly compared to different types of analyses such as coherence or phase analyses, between BP, RRI, sometimes including RE, and compared to RRI and SBP variabilities.

In healthy volunteers, it has been reported that causality analyses varied in a consistent manner with analyses of coherence between BP, RRI and RE in relation to age (Porta et al., 2014a), in paced breathing (Porta et al., 2012), in mental stress (Javorka et al., 2017), and in standing or lying position (Porta et al., 2014a; Javorka et al., 2017). It also appears that this type of analysis could be more informative because in both temporal or frequency domains, causal analyses make it possible to specify the nature of the relationship between RRI, SBP, RE or other physiological parameters studied (Faes et al., 2015b; Javorka et al., 2015; Porta et al., 2015; Song et al., 2015; Bose et al., 2017; Saleem et al., 2018). By studying baroreflex gain, we still note discrepancies with the study of causality (Porta et al., 2002; 2014b; Nollo et al., 2005; Javorka et al., 2017; Saleem et al., 2018). These two approaches could provide different information: unlike the gain which reflects the sensitivity of the baroreflex arc, the causality from SBP to RRI could reflect the presence of baroreflex feedback but not its sensitivity (Javorka et al., 2017). The dissociation of these two components of the baroreflex could allow study of two facets of this cardiovascular control that may be relevant in clinical perspectives. When these GC analyses were applied to patients, they make it possible to confirm certain mechanisms involved in certain diseases, such as in orthostatic intolerance and syncope (Faes et al., 2015b; Schiatti et al., 2015; Charleston-Villalobos et al., 2019; Reulecke et al., 2019). Consistently also with more traditional measurements, we have noted a decrease in the causality RE to RRI with age, probably related to the decrease in respiratory sinus arrhythmia; or the causality from RE to SBP, probably related to the decrease of cardiac function with age (Porta et al., 2014a); or even the preservation of a causality of RRI to SBP in the transplanted patient that indicates a contribution here of nonneural mechanisms (Porta et al., 2002). We can mention the study of Lenis et al. (2017) who used the GC method to separate the respiratory part responsible for RRI variability from the other mechanisms also involved. By comparing traditional heart rate variability (HRV) indices before and after decoupling, they showed that a large part of the interindividual differences in HRV was due to the different strengths among subjects, and they assumed that the calculation of HRV indices decoupled from respiration might add new relevant insights to the interpretation of HRV parameters. These studies revealed that causal analysis can effectively emphasize alterations or changes in cardiac regulation distinct from those identified by conventional tools. While the interpretation of causal relationships within the cardiovascular and respiratory systems remains somewhat hypothetical, these markers do not duplicate the information provided by traditional indices. In fact, they can sometimes exhibit a high sensitivity and a synergy between them that appears highly promising.

## 4 Methodological considerations and cautions

### 4.1 Quality of the MVAR model

**FIGURE 2:**
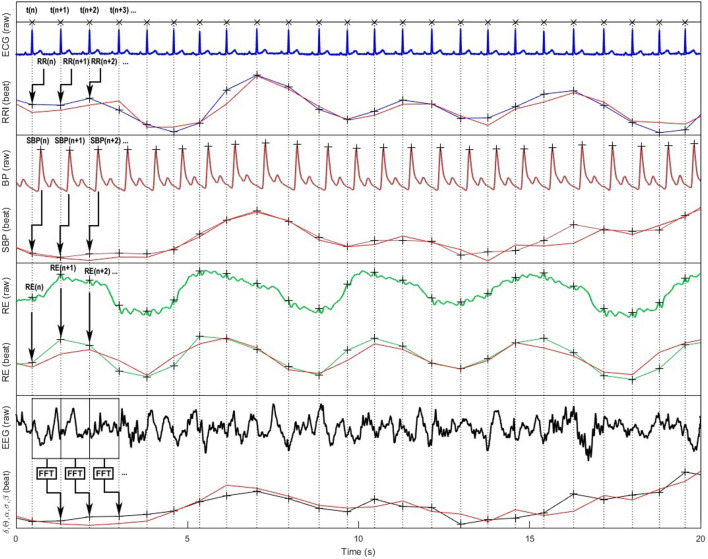
Representation of cardiovascular, respiratory and cortical raw signals and their corresponding beat-to-beat series according to the cardiac cycle are ; modelled signals used for Granger causality analyses are in red color. From top to bottom: ECG, RR intervals (RRI), continuous blood pressure (BP), systolic blood pressure (SBP), respiration (RE), electroencephalographic (EEG) signals, and the resulting standard δ, θ, α, β, γ spectral power bands (only α band is shown on the figure).

As described above, GC analyses are based initially on modelling the signals under consideration, i.e., here, RRI, BP and RE (Figure 2). These modelled signals must therefore be as faithful as possible to the original signals since they are used to calculate the causality indices linking them together (Granger, 1969; Porta et al., 2002). Model quality is generally estimated using the r-squared index which is the proportion of the variation in the predicted values of the signal compared to the true signal values, normalized by a value between 0 and 1; the closer the value is to 1, the better the model. Stationarity is an important prerequisite for the MVAR framework because the process characteristics must not change over time. In physiology, perfect signal stationarity is rarely achievable, but exclusion criteria are set before a signal is modeled, and the quality of the modelling is checked. This quality of the MVAR model, namely the fit index, must be considered before calculating causality indices. Empirically, a minimum value of 0.60 is often taken to validate sufficient model quality. Below this value, the modeled signal is not close enough to the original signal, and the resulting causality values should be disregarded.

In our experience, SBP signals are the best fits generally around 0.80–0.90, followed by RRI around 0.70–0.80 (Corbier et al., 2020). For respiration, we have noticed that the fit can be very variable (0.10–0.90) depending on the subjects and above all on the sensors used (nasal cannula, belt, ECG-derived respiration, etc.). The inherent differences in signal characteristics, such as sampling frequencies, can pose challenges when conducting a comprehensive analysis of causality. Consequently, considering the adequacy of signal alignment becomes a crucial factor in deciphering the outcomes of various studies.

It may be noted that the values of the quality index are never mentioned in articles dealing with interactions between RRI, SBP and RE using GC methods, nor is it ever specified whether certain data were discarded for these reasons. This may explain some of the variability in the results and the sometimes large standard deviations in certain causality indices in published studies, particularly those including respiration (Porta et al., 2013b).

### 4.2 Zero-lag effect

Causality in the classical Granger sense is defined as a cause taking place at a certain time before its effect, and the information about the process of this cause being unique, i.e., not present in any other process (Granger, 1969). Effects that might appear instantaneously are not considered in the classical method. Indeed, in fields such as physics or electronics, signals are sampled at a frequency such that there is always at least one delta of time above this frequency before a cause produces an effect. For cardiovascular and cardiorespiratory signals, the sampling frequency is set by the time interval between two heartbeats (around 1 s). However, certain regulatory mechanisms, such as the baroreflex due to the rapid response of the parasympathetic nervous system or the mechanical effects of respiration on stroke volume and cardiac filling, may be shorter than this time interval (Hyvarinen et al., 2008; Faes et al., 2010; Faes, 2014). As a result, cause and effect occur over a period of less than one cardiac cycle, indicating an instantaneous effect, i.e., a zero-lag effect, which must be considered at the risk of overlooking all the physiological processes involved. For this reason, the “extended” methods described above have been developed to take into account these instantaneous effects (Faes and Nollo, 2010; Porta et al., 2013b). When performing GC analyses for RRI, SBP and RE signals, it may also be useful to look specifically at the coefficients corresponding to this zero-lag effect in order to isolate the fast processes from the others.

In the study by Reulecke et al. (2019) for example, the authors clearly demonstrated the contribution of the extended method. In their example carried out during a tilt test on a healthy woman, the value of the causal link between SBP→RRI calculated with the extended method was higher than that obtained from the classic method due to the A (0) coefficient reflecting the rapid vagal response of the baroreflex taking place in the time interval of a heartbeat cycle. On the other hand, this instantaneous effect was not present for the inverse link, RRI→SBP. In the research conducted on syncope, it is noteworthy that the expansion of instantaneous analyses has proven particularly valuable in elucidating the dynamics of cardiovascular control disturbances (Schiatti et al., 2015; Saleem et al., 2018; Charleston-Villalobos et al., 2019). These studies demonstrate the benefits of using so called extended methods instead of the original one to study physiological signals in which sampling rate is determined by heartbeats.

### 4.3 Bivariate or trivariate models?

In many studies, particularly those focusing on the baroreflex, only the RRI and SBP signals are recorded and analyzed. However, for GC analyses applied to cardiovascular and respiratory systems, considering only these two signals can significantly modify the results (Porta et al., 2012; Bassani et al., 2013). Indeed, if these two signals interact in a closed loop, they are also both influenced by the respiratory signal. Porta et al. (2012) have studied this problem using simulated and real signals. In this study, the authors first explain using theoretical examples that the fact of not considering a signal z interacting with two other signals x and y, which themselves interact together, leads to an error in the calculation of the causal links between these two signals x and y. This error depends on the gain of the influence of signal z on signals x and y. Applied to RRI, SBP and RE signals, this means that if respiration has a significant effect on RRI and SBP signals, the joint variations in RRI and SBP will be falsely attributed to links between them if the respiratory signal is not included in the model. The results of the study by Porta et al. (2012) concerning real signals then clearly showed that not considering the respiratory signal when analyzing the causal links between RRI and SBP leads to an overestimation of the role of the baroreflex in these relationships. The authors even raised the question of the standard calculation of baroreflex sensitivity, which is based on the gain between variations in arterial pressure and the resulting variations in RRI (Parati et al., 1988; Bassani et al., 2012; Bassani et al., 2013) also showed in two studies investigating the causal links between RRI and SBP during anaesthesia, one including breathing and the other not, that the results and resulting interpretations could differ depending on whether the model was bivariate or trivariate (Figure 2).

Finally, without knowing the sufficient number of signals to be considered to model the cardiovascular and cardiorespiratory systems reliably, users should be aware that neglecting the influence of breathing on RRI and SBP could lead to erroneous results. This example unquestionably underscores the significance of adopting an integrative approach to studying the cardiovascular system, especially when utilizing such tools.

### 4.4 Different types of respiration sensors

Generally, the values of RRI and BP do not pose any particular problems in the context of measurements designed to analyze cardiovascular and cardiorespiratory regulation. In all cases, an ECG is recorded from which the time sequence of RRI is calculated. The only parameter to be monitored is the sampling frequency in order to obtain good precision in localizing R peaks; but nowadays this frequency is no longer limited by the storage capacities of the equipment as it once was, and we therefore no longer encounter undersampled signals. For BP, the equipment used is generally based on a finger photoplethysmographic arterial BP (Imholz et al., 1998), but it can also be measured with an intra-arterial catheter (see Supplementary Tables S1, S2). In addition to the precautions to be taken when choosing the sampling frequency, the experimenter must ensure that the position and height of the arterial pressure sensor are respected (Imholz et al., 1998). In all cases, whatever the equipment used, the type of signal recorded here is always an electrical voltage resulting from cardiac activity for the ECG signal (and therefore the RRI) and BP at artery level for the BP signal.

As can be seen in Supplementary Tables S1, S2, the same is not exact for respiratory measurement that may be taken from different places (oral or nasal flow or both, nasal capnogram, tidal movements of the chest or the abdomen, mathematical reconstruction from the ECG, etc.) resulting from or entailing various physiological mechanisms. The devices used for this purpose may be breathing belts with strain gauges or inductance placed on the thorax or abdomen, a nasal cannula, a thermistor, a full face mask including nose and mouth, or even an ECG from which the respiration is derived using the distortion of the QRS peak due to electrode motion and changes in the heart’s electrical axis synchronously to respiration induced thorax movements namely, ECG derived respiration (EDR) (Moody et al., 1985). Clearly, the principles of device measurement and signal collection sites can differ completely from one method to another. The shape of the signals and their temporality are affected, and this can be a problem when modeling the signal or calculating lag. For example, the shape of a respiratory signal arising from a chest or abdominal belt will differ from one subject to another, depending on the person’s breathing patterns, and may even be in phase opposition. Furthermore, how does the phase of a respiratory signal, whether measured by airflow at the mouth or reconstructed from ECG-derived respiration, come into play? To this must be added the fact that a sneeze, a sigh, even touching a measuring belt during a recording, or the approximations used in calculating the EDR will result in very significant artifacts which will not always be correctable and will potentially result in an inaccurate quality of the modelled respiration signal.

Porta et al. (2013b) had already mentioned the possible differences in physiological interpretation depending on the respiratory sensors used. Indeed, they demonstrated that the signals from a chest belt and EDR were similar in order to compare their results with other studies. Also, in another study in which cardiovascular interactions were calculated using the transfer entropy method, Gelpi et al. (2023) showed that causality values could be different depending on whether the RRI and SBP signals were conditioned by a respiratory signal from the chest belt or nasal capnogram. However, the form and temporality of the respiratory signals from different methods are not always comparable, and the difficulties in interpreting the causality results are still open to discussion (Porta et al., 2013b). It is therefore advisable to consider the type of equipment used for respiratory signal acquisition when interpreting causality results and comparisons with other studies should be made with caution. A study in which the different ways of measuring respiration and calculating causality are compared would be useful to clarify this methodological problem.

### 4.5 Available tools to study cardiovascular and respiratory interactions

In general, the tools available for calculating GC indices are aimed at people with a background in mathematics and programming. Below, three toolboxes (MATLAB and Python) were proposed and offered the most possibilities. Many other functions can be found on the internet, written in MATLAB, Python or R, but they are often restrictive such as being only suitable for bivariate analyses, for example,.

- The MVGC Multivariate Granger Causality (MATLAB): This free toolbox, developed at the Sackler Centre for Consciousness Science, University of Sussex, UK, provides MATLAB routines for efficient and accurate estimation and statistical inference of multivariate GC from time series data (Barnett and Seth, 2014).
- Luca Faes personal webpage (MATLAB): A comprehensive free library of functions in the field of causality, including extended versions of Granger’s analyses (Faes, 2021).
- The MVGC Multivariate Granger Causality (Python): This free toolbox is a simple multivariate GC Python tool rewritten from part of MATLAB MVGC toolbox (Qu, 2018).
- We also proposed one solution for nonprogrammer physiologists to analyze the causal links between RRI, BP and RE signals: CVRanalysis software is available for free at anslabtools. univ-st-etienne.fr (Pichot et al., 2023).

## 5 Clinical applications

Numerous studies have recently tested GC analysis in clinical applications. Two of these stand out from the rest in terms of the number of articles published: anaesthesia and orthostatic syncope or intolerance (Supplementary Table S2).

With regard to orthostatic syncope and intolerance, GC analyses have enabled to identify different mechanisms involved in healthy and pathological subjects (Schiatti et al., 2015; Charleston-Villalobos et al., 2019; Pernice et al., 2022b; Porta et al., 2023) as well as the kinetics of cardiovascular adaptation during a standing test (Reulecke et al., 2019). In particular, these studies showed that there was an increase in the causality index SBP→RRI with standing and that this index was higher in the supine position in patients compared to healthy subjects. Similarly, the causality values RRI→SBP were higher in patients than in healthy subjects in the supine position and during tilt tests. It was concluded that the increase in this index corresponded to an increase in baroreflex information flow resulting in an increase in sympathetic activity but also indicating impaired baroreflex function, and that the increase in this information flow in both directions in pathological subjects reflected the attempt to preserve sufficiently adequate cardiovascular regulation despite cardiovascular dysfunction. One study also showed that cardiorespiratory interactions were also affected in patients suffering from orthostatic intolerance (Charleston-Villalobos et al., 2019).

Concerning anaesthesia in an initial study, in which only the RRI and SBP signals were taken into account, Bassani et al. (2012) were able to show that during anaesthesia, there remained a causal link SBP→RRI due to baroreflex activity even when its sensitivity was collapsed. Two other studies that included the respiratory signal in the model confirmed that baroreflex was indeed involved in cardiovascular regulation during anaesthesia, but that the conventional methods used to validate measures of baroreflex sensitivity (RRI-BP squared coherence) overestimated its role because they integrated all the mechanisms acting on RRI and SBP indiscriminately (Porta et al., 2013a; Bassani et al., 2013). In addition, the authors observed that the type of ventilation or strategies of anesthesia had different effects on the SBP-RRI coupling and could be used by anesthetists to improve patient monitoring. Finally, these mathematical tools could be helpful to identify patients at-risk of hypotension during anesthesia (Dorantes Mendez et al., 2013) and those at-risk of atrial fibrillation after coronary artery bypass grafting, while the classical indices of variability and baroreflex were unable to do so (Bari et al., 2018).

In other clinical fields, GC analyses have shown interesting clinical results in various populations: changes in RE-SBP coupling in patients suffering from pre-eclampsia were reported (Riedl et al., 2010) and in RRI-SBP in Parkinson’s patients (Bassani et al., 2014). Interestingly, these causal analysis were related to quality of life in fibromyalgia patients (Zamunér et al., 2017) and can bring clinical information to aortic valve stenosis patients (Bari et al., 2023), patients with idiopathic pulmonary fibrosis (Santiago-Fuentes et al., 2022), pediatric cardiac patients (Rosol et al., 2022), heart failure populations (Radovanović et al., 2018), or patients with sleep apnea syndrome (Günther et al., 2022).

Many of these studies have consistently demonstrated that causal markers can effectively illuminate changes in cardiovascular and respiratory interactions that traditional indices often struggle to detect and bring information about physiological regulations. From a clinical perspective, the potential significance of these approaches lies in their ability to provide valuable markers for clinical assessment tools.

## 6 Perspectives

### 6.1 Central autonomic network and allostasia

Studying the interactions between the cardiovascular and respiratory systems provides valuable insights into the functioning of these systems, but the brain plays a key regulatory role, particularly in response to exteroceptive disturbances such as pain or any stimulation perceived as threatening. It is now well documented that through autonomic control, the brain controls the activity of major physiological systems. Here, GC is therefore a relevant approach integrating EEG or blood-oxygen-level dependent (BOLD) signals from functional magnetic resonance imaging (Figure 2), especially since recent work has shown that the brain, through interoception, receives a lot of visceral and somatic information that modifies its activity. In this view, a central autonomic network (CAN) as introduced in 1993 by Eduardo Benarroch, ensures the control of sympathetic and parasympathetic preganglionic neurons through a constant and integrative regulation of the functions of the different tissues and organs, maintaining homeostasis while ensuring responsiveness to external and internal changes (Benarroch, 1993). Functional magnetic resonance imaging studies have shown a close relationship between autonomic cardiac reactivity and cortical activities: whatever emotional, cognitive, sensorial or voluntary motor tasks applied in healthy subjects, a set of consistently activated brain regions, comprising amygdala, anterior and posterior insula and anterior cingulate cortices, supports cardiac reactivity and is supposed to form the CAN (Thayer et al., 2012; Beissner et al., 2013; Ruiz Vargas et al., 2016; Ferraro et al., 2022).

In this view, several studies applied GC between time series derived from EEG signals (typically δ, θ, α, σ and β frequency bands) and signals used to study cardiovascular and respiratory systems (such as RRI, SBP and RE) (Faes and Nollo, 2010; Faes et al., 2015a; Faes et al., 2016; Schulz et al., 2016; Valenza et al., 2016; Greco et al., 2019; Won et al., 2019; Orjuela-Cañón et al., 2020; Nardelli et al., 2021; Abdalbari et al., 2022). These studies aimed to document the central control on cardiovascular and respiratory systems and their interactions (Supplementary Table S3). First, applied between RRI and EEG signals, found bidirectional interaction between EEG signal and indices reflecting parasympathetic activity in healthy controls during sleep with a predominance from parasympathetic indices to EEG signal. However, a predominance from EEG to cardiac and autonomic parameters where frequently reported (Pardo-Rodriguez et al., 2021; Abdalbari et al., 2022; Pernice et al., 2022a; Orjuela-Cañón et al., 2022). Using high density EEG, Greco et al. (2019) observed a coupling predominance in fronto-central regions during emotional picture testing. It has frequently been shown that the power of rapid EEG oscillations evolves in the same trend as the LF and VLF variables (Faes et al., 2015a; Pardo-Rodriguez et al., 2021; Pernice et al., 2022a; Orjuela-Cañón et al., 2022). Applied during sleep, the brain-cardiac interaction seems to be decreased especially during slow wave sleep and paradoxical sleep (Orjuela-Cañón et al., 2020; Orjuela-Cañón et al., 2022; Pardo-Rodriguez et al., 2021; Günther et al., 2022); whereas Hartmann et al. (2021) showed a reciprocal interaction between cardiac and vascular autonomic parameters and cortical reactivity during sleep. In patients suffering from sleep diseases, this approach showed that a low causality in patients with sleep apnea was restored by continuous positive airway pressure therapy (Orjuela-Cañón et al., 2020). Finally, in clinical populations such as in schizophrenia, during sedation or in temporal epilepsies, these studies have made it possible to show alterations in the connectivity between the cardiovascular system and the brain which can be complex (Schulz et al., 2016; Won et al., 2019; Orjuela-Cañón et al., 2022). Also in the field of CAN studies, Yu et al. (2016) used Granger analyses applied to the BOLD time series arising from fMRI to study the functional connectivity of the respiratory neural network in chronic obstructive pulmonary disease patients and healthy controls. Although taken together, these data draw promising results regarding the relevance of this type of approach to better understand the repercussions of heart disease on cortical functions as well as of cortical functions on cardiac activity (Critchley and Harrison, 2013; Shivkumar et al., 2016).

### 6.2 Methodological perspectives

In the cardiovascular and cardiorespiratory fields, GC analysis has provided a complementary view to traditional analysis methods such as RRI or BP variabilities or cardiac baroreflex, which focus on the activity of the autonomic nervous system. GC methods have confirmed that cardiac baroreflex measurement alone is limited to explain the closed loop interrelationships between RRI and BP variations. Indeed, numerous studies have shown that the values of the BP→RRI causal links, representing baroreflex activity, were lower than those of the inverse link, RRI→BP. Studies have also shown the influence of respiratory movements on RRI and BP which is integrated in a very limited way into traditional analyses.

In fact, studies to validate the causal indices between RRI, BP and RE have been carried out using pharmacological blockades or stimulus tests such as the comparison between supine position and tilt test. All these studies have therefore essentially explored the autonomic part of these causality indices. However, it was clearly shown, for example, that autonomic pharmacological blockades did not alter the values of the BP→RRI causality indices, whereas one of the underlying relationships is parasympathetic activity. In that case, it was deduced that the value of the corresponding causality index did not provide information on the sensitivity of the baroreflex, but rather on the importance of recruitment of this reflex in regulation, and that the existence of this causal link was a prerequisite for reliable assessment of the baroreflex. Other mechanisms have been suggested as contributors of causality, including mechanical influences such as Starling and the Windkessel effects in RRI→BP coupling that mechanically relate changes in intracardiac pressure and stroke volume to blood pressure or even mechanical effects of respiratory on intrathoracic pressure (RE→BP) and on the sinus node tissue (RE→RRI). More complex and hypothetical neural influences emerge that could contribute to these couplings such as influences of respiratory centers and vagal efferent activities, or the activation of cardiopulmonary reflexes explaining bidirectional coupling between respiratory and cardiac changes. Studies specifically exploring the relationship between these mechanisms and GC in isolation are still lacking, and they could give greater weight to the results and interpretations of the calculated indices, particularly for their use in clinical applications or morbimortality prediction.

There is, however, a methodological difficulty to be circumvented, which forces us to make compromises in the analyses. The frequency bands of interest for EEG are much higher than those for RRI signals. EEG sampling frequencies are generally at least 256 Hz, and the resulting brain rhythms (delta, theta, alpha, beta and gamma spectral power bands) range from 1–120 Hz; whereas for RRI signals the time interval between individual heartbeats corresponds to a sampling frequency of the order of 1 Hz. In this case, it is not easy to calculate a causal link between the two types of signals or to interpret the results physiologically. Some authors have used GC methods to study heart-brain interactions based on RRI and EEG signals (Faes et al., 2015a; Greco et al., 2019; Orjuela-Cañón et al., 2020). However, there remains a wide field of exploration and clinical application in this area which cannot be achieved without improving investigative methods.

### 6.3 Clinical and morbimortality prediction

Cardiovascular variability indices have been widely democratized, first in the field of cardiology with tabletop ECGs and especially Holter systems, then more widely into the fields of sports and wellness with RRI monitors and connected watches (European Society of Cardiology and the North American Society of Pacing and Electrophysiology, 1996). Indices of parasympathetic and sympathetic nervous system activity are commonly considered in the monitoring of cardiac populations (Manresa-Rocamora et al., 2021), the severity of autonomic neuropathy in diabetic patients (Žnidarič et al., 2023) or maturation of premature babies (Patural et al., 2008), for example, as well as in sports training to optimize sessions and avoid overtraining (Plews et al., 2013). Certain indices of RRI variability or baroreflex related indices are well known to be good predictors of all-cause mortality or the occurrence of cardiovascular or cerebrovascular events in pathological or healthy populations (Tsuji et al., 1994; Dekker et al., 1997; La Rovere et al., 1998; Berger et al., 2022).

As described above, the study of the causal links between RRI, BP and RE using Granger analysis provides complementary information to so called traditional methods when applied to protocols carried out in both healthy and pathological subjects. These analysis methods could therefore be used to monitor the training of athletes or the exercise rehabilitation of pathological patients. These complementary indices could make it possible to focus on the effects of training on much more precise mechanisms, as suggested by the study by de Abreu et al. (de Abreu et al., 2022), thus helping to better target the type of exercise used in the training or rehabilitation program. Moreover, to our knowledge, no cohort study has yet demonstrated the usefulness of these indices in predicting the occurrence of diseases, events or mortality (Schulz et al., 2013; Müller et al., 2016). It would therefore be interesting to test the predictive capacities of causality indices in cohorts of healthy or cardiac subjects, for example,. The results could perhaps highlight other pathophysiological mechanisms linked to morbidity and mortality than reduced autonomic nervous system activity or baroreflex sensitivity.

## 7 Conclusion

Cardiovascular and cardiorespiratory control is formed by multi-interacting mechanisms and systems, and GC analysis is particularly suitable for identifying these interactions. Clinical and experimental studies support that causality analysis provides nonredundant information with respect to more traditional means obtained using bivariate, trivariate or multivariate approaches. It appears that neural and mechanical mechanisms are probably responsible for these interactions between RRI, BP and RE, including Windkessel and Starling effects, Cushing reflex, autonomic control and other more indirect mechanisms to be defined. Their clinical interests in understanding and predicting diseases must be specified and these issues merit further investigation. Finally, Walter Cannon laid the foundations of an experimental and theoretical framework characterizing the general response of the body to external stresses or during disturbances of homeostasis, speaking of “emotional stress” in 1935 (Cannon, 1935). Afterwards, the concept of allostasis that incorporates changes of steady state level – “stability through change” - was proposed (Sterling and Eyer, 1988). These mathematical tools appear helpful for gaining a deeper understanding of the various interactions at play, especially in discerning the contribution of the CAN within these allostatic regulations.

## Supplementary material

SUPPLEMENTARY TABLE S1 Studies on cardiovascular and respiratory interactions based on Granger causality analysis in bivariate, trivariate or multivariate approaches in heathy volunteers.

SUPPLEMENTARY TABLE S2 Studies on cardiovascular and respiratory interactions based on Granger causality analysis in bivariate, trivariate or multivariate approaches in clinical populations.

SUPPLEMENTARY TABLE S3 Studies on cardiovascular and respiratory interactions based on Granger causality analysis in multivariate approaches including electroencephalography signals. *δ,* θ, α, *σ*, β and γ represent the EEG spectral power bands between 0.5-4 Hz, 4-8 Hz, 8-12 Hz, 12-16 Hz, 16-30, and 30-100 Hz which can vary depending on the studies.
